# Space medicine: gut microbiome of hardy species is a potential source to counter disorders during space travel

**DOI:** 10.2144/fsoa-2023-0060

**Published:** 2023-05-16

**Authors:** Ruqaiyyah Siddiqui, Adel B Elmoselhi, Naveed Ahmed Khan

**Affiliations:** 1 College of Arts & Sciences, American University of Sharjah, University City, Sharjah, 26666, UAE; 2 Department of Medical Biology, Faculty of Medicine, Istinye University, Istanbul, 34010, Turkey; 3 College of Medicine, University of Sharjah, University City, Sharjah, 27272, UAE

**Keywords:** metabolomics, screening, target/lead discovery

## Abstract

It is proposed that gut microbiome of species like cockroaches may offer a potential source of novel mechanisms/molecules that can be translated into humans to safeguard astronauts against stressors of the space environment during deep space exploration missions.

There has been a great deal of interest in long-duration space missions ever since hundreds of astronauts first travelled into space in 1961. This is demonstrated by the latest Global Exploration Roadmap study by 20 country space agencies [1]. It outlines a strategy for increasing space activity and sets the goal of populating Mars by 2050 in addition to conducting frequent manned spaceflights [2]. This is despite the knowledge that microgravity, exposure to radiation, isolation and confinement may lead to an imbalance in physiological reactions, as well as dysbiosis of the gut microbiota, leading to a negative impact on an astronaut’s health and performance, and space travel may trigger a variety of stressors [3]. Increased radiation exposure and associated immunological dysregulation have been directly connected to space missions and have been highlighted in research over the past few decades [4–7]. Immune system dysregulation can result in related disorders as immunity is interconnected with all other physiological systems. Thus, it is necessary to find molecules or mechanisms with the potential for translation in order to boost the immune system during space travel. Here, we discuss the stressors that space travel may bring about, which are likely interconnected with gut microbial dysbiosis and propose that, in order to augment human health and wellbeing, the defense and protection mechanisms (likely their gut microbiome is a contributory factor) of ‘hardy and resilient’ species like crocodiles, cockroaches and tardigrades should be investigated. We suggest that the development of pre/pro/postbiotics from these unique species may be utilized for astronauts to prevent or restore gut microbial dysbiosis and thus will be valuable for their health and performance.

## What are the unique attributes of hardy species?

Even though radiation does not just occur in space, human exposure to radiation in space is significantly higher than on Earth. For instance, compared with 2.4 millisievert on Earth, humans would receive between 400 and 900 millisievert annually in interplanetary space. A recent study predicted that mice exposed to radiation during a Mars expedition would have a twofold increased risk of acquiring cancer [8]. Even with the development of radiation protection strategies such as creams, clothing, glass and others, the incidence rate of cancer in humans can be as high as 50%, just with exposure of 2.4 millisievert on Earth (although many factors including lifestyle, diet, environmental settings, are also contributory factors).

The ability of species like cockroaches, crocodiles and tardigrades to withstand high radiation levels, live in filthy environments, consume food that is contaminated with a plethora of microbes, and frequently be exposed to heavy metals is fascinating to investigate, especially as these species are seldom described to develop diseases such as cancer [9]. For instance, tardigrades, can withstand extreme radiation, being frozen for more than a year, dehydration, survival at temperatures varying from zero to 100°C and six-times more pressure than at the ocean’s bottom. These species are currently regarded as one of the most resilient animals on Earth [10,11]. It is amazing how well they protect DNA from radiation damage. Given their remarkable properties, tardigrades were launched into low-Earth orbit in 2007, and survived exposure to vacuum and cosmic radiation and even made it through lethal UV radiation levels [12]. While the detailed molecular mechanisms are not known, this is partially due to a protein called Dsup (damage suppressor), which has the ability to: physically protect DNA without impairing its function; and remove reactive oxygen species, increasing radiation resistance [13]. The observation that overexpression of Dsup protected human cells from radiation damage is encouraging and presents a wealth of potential [13]. Compared with the controls, cells expressing Dsup showed up to 50% reduced DNA damage from x-rays. A number of protective genes and/or enzymes that repair DNA and/or neutralize reactive oxygen species, including MRE11, were also found. These findings open up a possible path toward improving human radiation resistance during space missions by genetically engineering Dsup transfer.

One of the few creatures that can endure high radiation levels is the cockroach, such as the 10,000 rads produced by an atomic bomb. One thousand feet from the site of the Hiroshima atomic bomb drop, cockroaches were discovered to be thriving. Cockroaches are recognized to have the ability to endure high levels of radiation, and it was previously suggested that insects like cockroaches would be ideal biological organisms to study in space [14,15]. Some species, like crocodiles, are regularly exposed to radiation (from the sun), heavy metals and other cancer causing substances [14,16]. It is hypothesized that these species may have evolved strategies to counter cancer development or developing other diseases. In addition, *H. sapiens* is indeed one species among many millions of others. While other species have lived on the globe for millions of years, we are only a somewhat recent arrival. It is puzzling how species like the crocodile can endure carcinogenic conditions that are harmful to humans and still live up to almost a century. Very likely, these species have developed defenses against the emergence of cancer. These results have two plausible explanations: such creatures have evolved a robust immune system; and/or the GI tract and oral microbiota of the aforementioned species produce unique biomaterials. These observations make it imperative to examine these mechanisms and/or sequester their bioactive compounds for the health and welfare of humans. Many studies show that the microbiome plays a crucial part in regulating the host’s health and has a vital role in influencing astronaut health while in space. Therefore, it is necessary to create methods to retain the proper ratios of bacterial species in the gut for optimal health. These methods should also be tested in analog flights, as well as *in vivo* and *in vitro* models of microgravity/space travel that are based on the ground.

It is necessary to have a deeper comprehension of the unique compounds produced by fascinating species like crocodiles and cockroaches, as well as their detailed and specific effects on aging, cancer, cellular senescence and general human health [16]. A recent study evaluated the composition of the crocodile gut bacterial species and it was revealed that several bacterial species including *Actinobacteria, Proteobacteria, Bacteroidetes, Firmicutes* and *Deinococcus-Thermus* were present [17]. Additionally, LC–MS was used to clarify the types of molecules found in the bacterial metabolites found in the crocodile gut. The analysis revealed a large number of molecules, though many remained unidentified and may therefore be novel. Recent research has shown how these species molecules have remarkable anticancer and antibacterial properties [9,17]. In another study, *Serratia marcescens, Escherichia coli, Klebsiella species, Bacillus species* and *Streptococcus species* were among the bacteria that were isolated from the cockroach gut microbiota [18]. Conditioned medium containing gut bacterial metabolites was then evaluated, and these gut bacterial metabolites were found to have bactericidal and inhibitory effects [18]. Furthermore, we suggest that the microbiome of tardigrades should be investigated for anti-bacterial/beneficial effects. Following these investigations, to carry out metabolomics and proteomics investigations to find possible bioactive compounds, LC–MS will be a particularly useful method. Molecular identification can be determined using nuclear magnetic resonance spectroscopy in conjunction with this. To protect astronauts from the stresses of the space environment, these molecules could subsequently be utilized as prebiotics or postbiotics and evaluated *in vivo* using microgravity models like the hindlimb unloading model [19]. The notion of experimentation with cockroaches in the microgravity environment, to understand their ability to endure radiation in space is worthy of investigation. Such innovative research will open the door for our search for preventative and/or therapeutic solutions to potential hazards brought on by the rising number of deep space exploration missions. Therapies with space travel in mind should be devised, and these can be achieved by integrating existing understanding of the gut microbiota from clinical research of relevance [20].

## References

[1] Laurini K, Piedboeuf JC, Schade B, Matsumoto K, Spiero F, Lorenzoni A. The global exploration roadmap IAC-11 B, 3. (2018). https://fiso.spiritastro.net/telecon13-15/Laurini_12-11-13/Laurini_12-11-13.pdf

[2] Pelton JN. Spaceplanes, space tourism and private space habitats. In: Space 2.0. Springer, Cham, Switzerland, 157–168 (2019).

[3] Siddiqui R, Akbar N, Khan NA. Gut microbiome and human health under the space environment. J. Appl. Microbiol. 130, 14–24 (2021). 32692438 10.1111/jam.14789

[4] Cogoli A. The effect of space flight on human cellular immunity. Environ. Med. 37, 107–116 (1993). 12211252

[5] Sonnenfeld G. Effect of space flight on cytokine production. Acta Astronaut 33, 143–147 (1994). 11539514 10.1016/0094-5765(94)90119-8

[6] Guéguinou N, Huin-Schohn C, Bascove M Could spaceflight-associated immune system weakening preclude the expansion of human presence beyond Earth's orbit? J. Leukoc. Biol. 86, 1027–1038 (2009). 19690292 10.1189/jlb.0309167

[7] Konstantinova IV, Rykova MP, Lesnyak AT, Antropova EA. Immune changes during long-duration missions. J. Leukoc. Biol. 54, 189–201 (1993). 8371048 10.1002/jlb.54.3.189

[8] Cucinotta FA, Cacao E. Non-targeted effects models predict significantly higher mars mission cancer risk than targeted effects models. Sci. Rep. 7, 1832 (2017). 28500351 10.1038/s41598-017-02087-3 PMC5431989

[9] Jeyamogan S, Khan NA, Siddiqui R. Animals living in polluted environments are a potential source of anti-tumor molecule(s). Cancer Chemother. Pharmacol. 80, 919–924 (2017). 28795217 10.1007/s00280-017-3410-x

[10] Petrescu-Mag IV. Tardigrades traveled to space and survived. Extreme Life, Biospeology and Astrobiology 8(2), 64–66 (2016).

[11] Sloan D, Alves Batista R, Loeb A. The resilience of life to astrophysical events. Sci. Rep. 7(1), 5419 (2017). 28710420 10.1038/s41598-017-05796-x PMC5511186

[12] Jönsson KI, Rabbow E, Schill RO, Harms-Ringdahl M, Rettberg P. Tardigrades survive exposure to space in low Earth orbit. Curr. Biol. 18(17), R729–R731 (2008). 18786368 10.1016/j.cub.2008.06.048

[13] Hashimoto T, Horikawa DD, Saito Y Extremotolerant tardigrade genome and improved radiotolerance of human cultured cells by tardigrade-unique protein. Nat. Commun. 7, 12808 (2016). 27649274 10.1038/ncomms12808 PMC5034306

[14] Wharton DR, Wharton ML. The effect of radiation on the longevity of the cockroach, *Periplaneta Americana*, as affected by dose, age, sex and food intake. Radiat. Res. 11(4), 600–615 (1959). 13844254

[15] Sullivan WN, Schechter MS, Dutky SR, Keller JC. Monitoring electrophysiological responses of cockroaches for space research. J. Econ. Entomol. 55(6), 985–989 (1962).

[16] Siddiqui R, Maciver S, Elmoselhi A, Soares NC, Khan NA. Longevity, cellular senescence and the gut microbiome: lessons to be learned from crocodiles. Heliyon 14, e08594 (2021). 10.1016/j.heliyon.2021.e08594 PMC8688568 34977412

[17] Khan NA, Soopramanien M, Maciver SK, Anuar TS, Sagathevan K, Siddiqui R. *Crocodylus porosus* gut bacteria: a possible source of novel metabolites. Molecules 26(16), 4999 (2021). 34443585 10.3390/molecules26164999 PMC8398445

[18] Akbar N, Siddiqui R, Iqbal M, Sagathevan K, Khan NA. Gut bacteria of cockroaches are a potential source of antibacterial compound(s). Lett. Appl. Microbiol. 66(5), 416–426 (2018). 29457249 10.1111/lam.12867

[19] Morey-Holton E, Globus RK, Kaplansky A, Durnova G. The hindlimb unloading rat model: literature overview, technique update and comparison with space flight data. Adv. Space Biol. Med. 10, 7–40 (2005). 16101103 10.1016/s1569-2574(05)10002-1

[20] Le Roy CI, Kurilshikov A, Leeming ER Yoghurt consumption is associated with changes in the composition of the human gut microbiome and metabolome. BMC Microbiol. 22, 1–2 (2022). 35114943 10.1186/s12866-021-02364-2 PMC8812230

